# Effect of nose twitching on the pupillary dilation in awake and anesthetized horses

**DOI:** 10.3389/fvets.2024.1412755

**Published:** 2024-07-04

**Authors:** Carlota Emilia Mascaró Triedo, Sahra Karar, Maha Abunemeh, Karine Portier

**Affiliations:** ^1^VetAgro Sup, CREFAC, Université de Lyon, Marcy l’Etoile, France; ^2^Centre de Recherche en Neurosciences de Lyon, INSERM, CRNL U1028 UMR5292, Trajectoire, Université Claude Bernard Lyon 1, Bron, France

**Keywords:** horse, pain assessment, anesthesia, analgesia, pupillometry, pupillary dilation

## Abstract

Pupillometry is used in humans to monitor pain, nociception and analgesia. This single-center, non-randomized, non-blinded intervention trial, evaluated the effect of nose twitching on the pupil size in awake, sedated, and anesthetized horses. Pupil height (H) and length (L) were measured before (Be) and after (Af) nose twitching in fourteen non-painful adult awake horses (T0). The percentage of variation (PSV) was calculated (PSVTn = [(TnAf-TnBe)/TnBe]*100). Measurements were repeated (Tn) after acepromazine (0.04 mg kg^−1^ IV) (T1), romifidine (0.04 mg kg^−1^ IV) (T2), morphine (0.1 mg kg^−1^ IV) (T3), after anesthesia induction with diazepam (0.05 mg kg^−1^ IV) and ketamine (2.2 mg kg^−1^ IV), at the time the horse was placed on the operating table (T4) and when the expiratory fraction of sevoflurane was 2% (T5). HAf vs. HBe, LAf vs. LBe as well as PSVH vs. PSVL at each time were compared with a Mann–Whitney Wilcoxon test. The PSVL and PSVH, as well as HBe and LBe over time were compared with the Skillings-Mack test followed by a Wilcoxon test for paired data to make pairwise comparisons (Tn + 1 vs. Tn). In non-sedated horses (T0), the application of the nose twitch induced a significant increase in pupil length (LT0Be: 17.09 [16.05; 19.67] mm versus LT0Af: 19.52 [18.74; 21.40]) mm (*p* = 0.004). Thirty minutes after acepromazine administration (T1), nose twitching induced a significant increase in pupil length (LT1Be: 16.45 [14.80; 18.66] mm versus LT1Af 18.31 [17.20; 20.52] mm) (*p* = 0.016) and height (HT1Be: 8.44 [5.68; 12.04] mm versus HT1Af: 11.09 [7.97; 14.3] mm) (*p* < 0.001). PSVHT1 was significantly greater than PSVLT1 (*p* = 0.025). PSVH was higher at T1 than at T0 (*p* = 0.04). It was also significantly higher at T1 than at T2 (*p* < 0.001). Romifidine induced mydriasis (HT2Be 16.95 [14.73; 18.77] mm versus HT1Be 8.44 [5.68; 12.04] mm) (*p* < 0,001) (LT2Be 19.66 [18.45; 20.41] mm versus LT1Be 16.45 [14.80; 18.66] mm) (*p* < 0.001). The results suggest that nose twitching induced a pupillary dilation in the awake horse. This effect was potentiated after the administration of acepromazine but disappeared after the administration of romifidine.

## 1 Introduction

Pain can only be expressed by individuals who are alert and able to speak. It is therefore difficult to measure in babies, debilitated and anesthetized individuals. In animals, pain must be assessed by humans, which leads to a bias in interpretation due to the sensitivity of the observer (1).

The subjectivity of pain perception highlights the need for objective tools to assess it, particularly in non-communicative subjects. Pain scales based on behavior, physiological parameters or facial expression have been developed, but remain subject to the subjective interpretation of the observer (1).

Devices have been developed to more objectively measure conscious and unconscious pain (nociception) in humans and animals. They record physiological variables, which are then integrated by algorithms to improve the objectivity of pain measurement (2).

Among these techniques, pupillometry has recently been shown to be an objective and reliable monitor of the level of antinociception in humans, allowing opioid administration to be reduced (2, 3). Pupillometry has also been used to monitor pain in awake patients (4–6).

Pupillometry has many advantages. It is non-invasive, easy to apply, inexpensive to purchase, it does not require advanced analyses, and is reproducible. In addition, data can be obtained in real-time (7).

The principle is based on the fact that painful stimulation reliably causes pupil dilation. This mydriasis occurs simultaneously with changes in the balance of the autonomic nervous system. The regulation of pupillary diameter results from the action of antagonistic muscles (the sphincter muscle and the dilator muscle) present in the iris. The former is modulated by the cholinergic fibers of the parasympathetic system and the latter by the adrenergic fibers of the sympathetic system (8–10).

Furthermore, the amplitude of the pupillary dilatation reflex (PDR) is proportional to the intensity of the nociceptive stimuli. This can be observed in awake and anesthetized patients (3–5). However, some drugs can affect the PDR because they alter the pupil response to a painful stimulus (9, 11). For example, opioids increase parasympathetic activity, which causes contraction of the circular sphincter of the iris and thus induces miosis in humans. In the event of a painful or nociceptive stimulus, they prevent the transmission of the nociceptive signal, thereby inhibiting the PDR (3, 11, 12). Among anesthetic agents, alpha-2-agonists prevent mydriasis in dogs by inhibiting the action of the iris dilator muscle (13, 14). However, alpha-2 agonists may induce mydriasis in several animal species, by activation of alpha-1 receptors in the dilator muscle, although they are not very specific. The sensitivity of the receptors varies according to the species (there are also receptors on the sphincter muscle, in smaller quantities) (11, 15). Systemic ketamine and atropine have a mydriatic effect (16).

To our knowledge, pupil dilation in reaction to a painful/nociceptive stimulus has not yet been studied in animals. In horses, pupil size measurement can be performed under general anesthesia because the eye remains in a central position. Pupillometry could be of particular interest in adapting intraoperative analgesia in this species. Indeed, pain or excessive analgesics administration such as opioids, can have deleterious effects, such as gastro-intestinal ileus (17–19) and can lead to poor recovery, which is the period most at risk during anesthesia in horses (20).

This study aimed to evaluate the effect of the application of a nose twitch (considered as a painful stimulus) to the horse’s upper lip, on pupil size in awake and anesthetized horses. We hypothesized that this stimulus induces pupil dilation in awake horses, but that this dilation is affected by some molecules usually included in general anesthesia protocols.

## 2 Materials and methods

This single-center, non-randomized, unblinded experimental study was carried out at the equine clinic Clinequine (VetAgro Sup’s equine university hospital). The study was approved by the Ethical Committee of the National Veterinary School of Lyon (Number 2234, April 12th, 2022). Written consent was obtained from each owner before their horse was included in the study.

### 2.1 Animals

**Figure 1:**
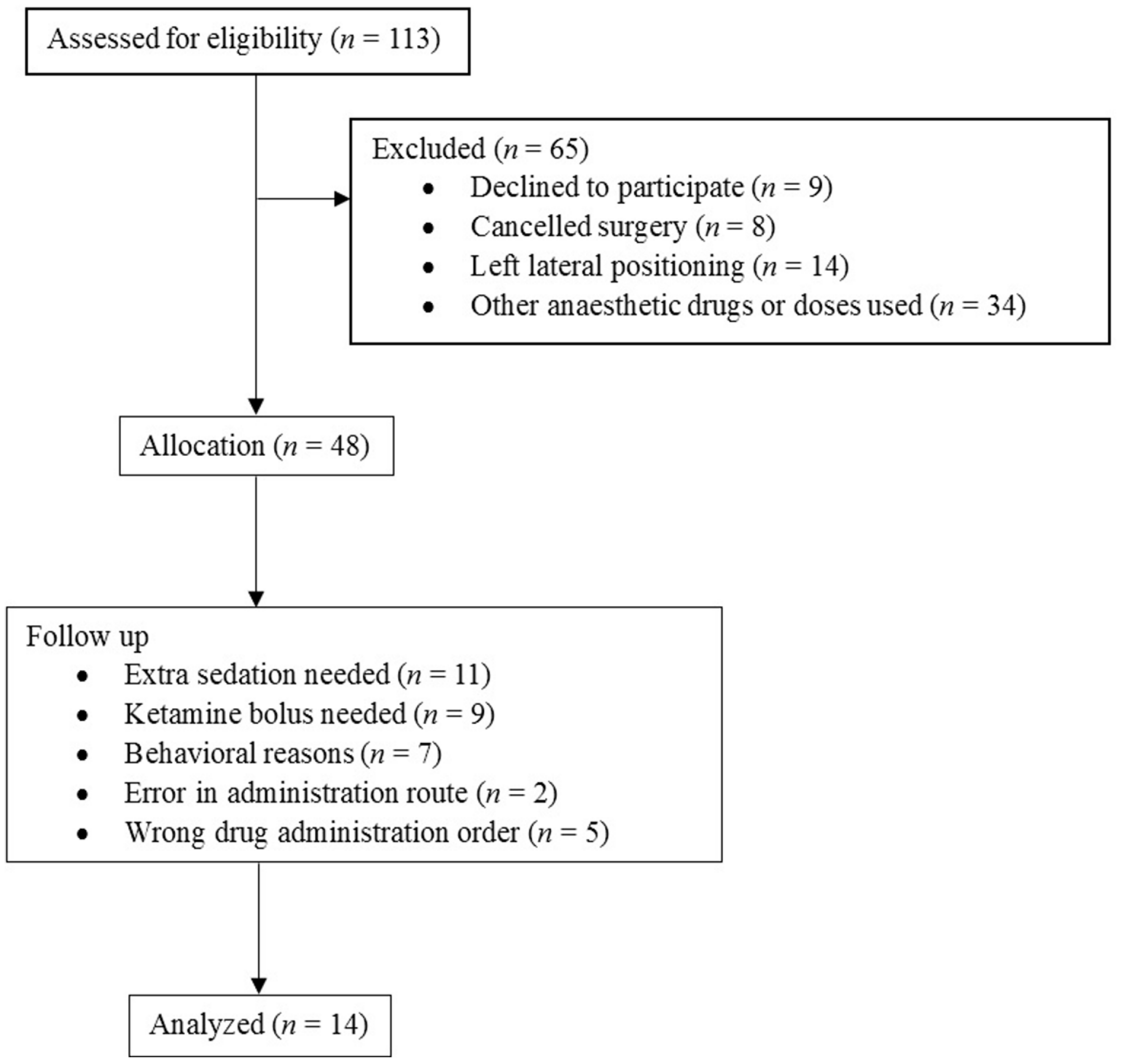
Flow diagram. Inclusion and exclusion flow diagram of 113 horses presented into the veterinary hospital for elective surgery.

The target population was horses over 2 years of age, free of acute or chronic pain, admitted to the hospital for elective surgeries under general anesthesia between 1 August 2022 and 30 June 2023. Horses had to be free of ocular disease and not medicated at the time of admission. Horses were required to behave in such a way as to allow the application of the nose twitch to the upper lip without risk to either the horse or the handlers (Figure 1).

The study was carried out in awake horses and at each stage of the general anesthesia protocol. The pupil size was measured with a pupillometer previously modified to fit the horse’s eye (AlgiScan®, IDMed, Marseille, France). For each evaluation, two measurements were taken. The first measurement (Be) was taken before the nose twitch was applied. The nose twitch was then applied to the horse’s upper lip and the string rotated until it stopped, and a second measurement (Af) was taken immediately. The nose twitch was then removed. These two measurements were compared to assess whether the application of the nose twitch caused a variation in pupil size. The same nose twitch was used in the same way by the same person for all the horses included in the study. Also, for each measurement, a photo of the pupillometer’s screen was taken so that a blinded external assistant could check the measurement.

To assess the influence of the anesthetics and analgesics used during the anesthesia protocol on pupil size, the evaluation previously described was repeated after the administration and onset of action of each molecule. Each horse, measured awake, was its control during the study. For each horse, the same eye was measured during the study. To facilitate access to the eye, the left eye was preferentially chosen because the right one was near the wall of the induction box to assist lying down, which made the picture difficult. Horses undergoing surgery requiring left lateral decubitus on the operating table were excluded.

### 2.2 Procedure

**Figure 2:**
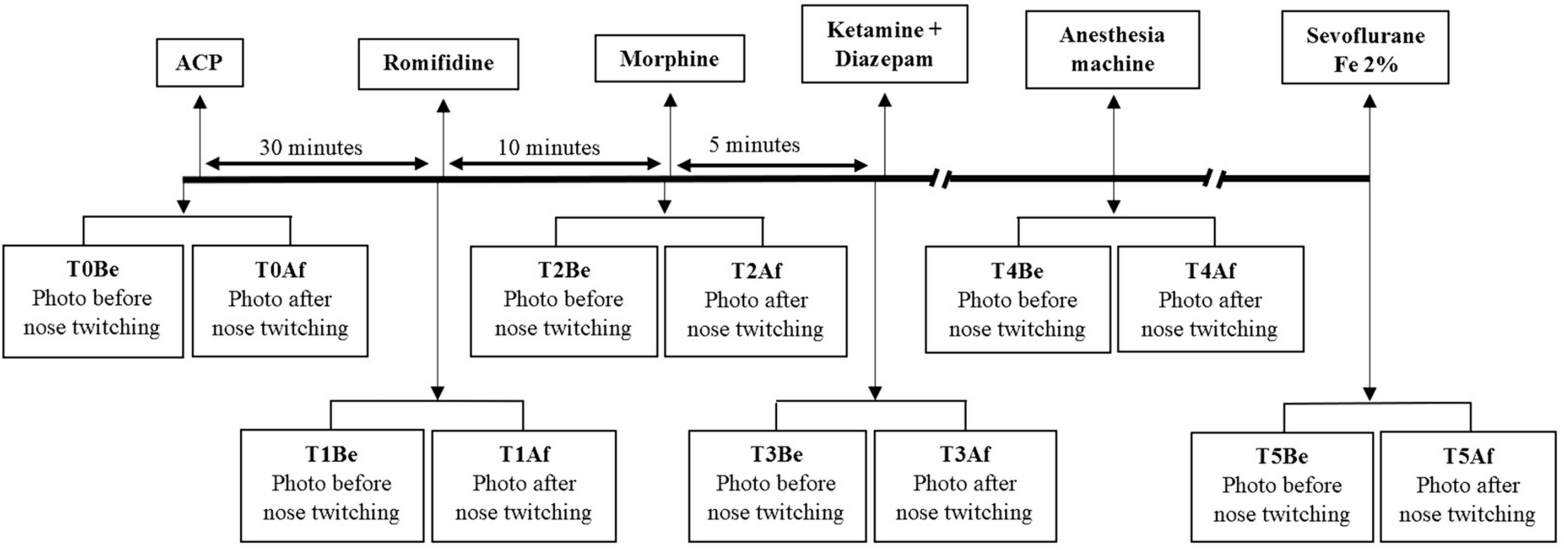
Study design. Study timeline of 14 horses included in the study showing the interventions. A first measure (T0) was taken. Premedication with acepromazine (ACP) 40 μg kg^−1^ IV was done and 30 min later, a second measure was taken (T1). Then, romifidine 40 μg kg^−1^ IV was administered and 10 min later, a third measure was taken (T2). After that, morphine 0.1 mg kg^−1^ IV was administered and 5 min later, a fourth measure was taken (T3). Then, the anesthesia induction was done with the administration of ketamine 2.2 mg kg^−1^ IV and diazepam 0.05 mg kg^−1^ IV. Once the horse was connected to the anesthetic machine, a fifth measure was done (T4). After stabilization of a sevoflurane expired fraction of 2%, a last measure was taken (T5).

For each horse, six time points (Tn with *n* = 0 to 5) were observed, each representing two pupil measurements: before (TnBe) and after (TnAf) the nose twitch was applied (Figure 2).

The values measured for height (H) and length (L), before (Be) and after (Af) the application of the nose twitch at each stage of anesthesia (Tn) were used to calculate a percentage in pupil size variation (PSV) in length (PSVLTn) and in height (PSVHTn). The PSV was calculated, for example for the height H, according to the following formula:


$$\mathrm{PSVHTn}=\frac{\mathrm{HTnAf}-\mathrm{HTnBe}}{\mathrm{HTnBe}}\times 100$$

A 14-gauge catheter (BD Angiocath) was placed in the jugular vein for every horse enrolled in the study. An initial assessment was carried out on the awake horse (T0) in its hospital stall. Each horse was then premedicated with intravenous (IV) acepromazine (0.04 mg kg^−1^). Thirty minutes later, the horses were taken to the induction stall and the pupillary dilation (PD) evaluation was performed (T1). The horses were then sedated with intravenous romifidine (0.04 mg kg^−1^). Ten minutes later, the PD was assessed again (T2). Morphine (0.1 mg kg^−1^ IV) was then slowly administered. The PD was assessed again 5 min after morphine administration (T3). Anesthesia was induced with diazepam (0.05 mg kg^−1^ IV) and ketamine (2.2 mg kg^−1^ IV). Once the horses were lying down, the trachea was intubated and the animals were placed on a padded table and connected to an anesthesia machine in the operating room. As soon as the horse was connected to the anesthesia machine, a new PD assessment was performed (T4).

Mechanical lung ventilation was started immediately (6 breaths per minute, at a tidal volume of 10 mL kg^−1^ and a maximum inspiratory pressure of 30 cmH_2_O) and adjusted during anesthesia to maintain a partial pressure of mean expired carbon dioxide (P_E_CO_2_) between 35 and 45 mmHg. Sevoflurane (in 100% oxygen) was used to maintain anesthesia on a surgical plane. The inspired fraction of sevoflurane (FiSevo) was adjusted during a stabilization period until the expiratory fraction of sevoflurane (FeSevo) reached 2%. At this point, a final PD assessment was performed (T5). As soon as the last PD measurement was made, a continuous constant rate infusion of romifidine (0.04 mg kg^−1^ h^−1^) was administered concomitantly with the sevoflurane. Doses of ketamine (0.5 mg kg^−1^ IV) were administered if the animal showed spontaneous movement or nystagmus. Horses that required a ketamine bolus during the PD measurements were excluded from the study. Ringer lactate was administered intravenously during anesthesia at a rate of 10 mL kg^−1^ h^−1^.

Anesthetic monitoring included heart rate (HR) and rhythm, invasive blood pressure (IBP) measured using a catheter placed in the facial artery, respiratory rate (RR), tidal volume (Vt), pulse oximetry, P_E_CO_2_, FeSevo and FiSevo. Depth of anesthesia was monitored by assessment of clinical signs and absence of spontaneous movement. After the last measurement, dobutamine could be administered intravenously at a rate of 2 to 8 μg kg^−1^ min^−1^ using a syringe pump to maintain mean arterial pressure around 60 mmHg to 70 mmHg. Arterial blood gases were analyzed every hour. At the end of the procedure, the animals were disconnected from the respiratory circuit, transferred to the recovery stall, and placed in lateral decubitus with the lower forelimb pulled forward. Recovery was unassisted. Romifidine (0.02 mg kg^−1^ IV) was administered during recovery if necessary.

### 2.3 Statistical analysis

Statistical analyses were performed using R software (version 2023.06.1-524). The normality of our data was tested using the distribution of differences for each pair of data (before and after the application of the nose twitch for each time Tn). The data did not follow a normal distribution and were treated by non-parametric tests. Differences were considered statistically significant if *p* < 0.05. The figures are presented in the form of the median and the interquartile range [Q1; Q3].

A power calculation was performed to calculate the minimum sample size required to have an 80% chance of identifying a 25% variation in pupil height and a 15% variation in pupil length. This calculation indicated that 14 horses were required.

For each time Tn, the sizes before and after the application of the nose twitch and the percentages of pupil size variation (PSV) in height and length, were compared using the Mann–Whitney Wilcoxon test for paired data.

To compare the effect of the molecules on the pupil size, we compared PSV in height (PSVHTn) and in length (PSVLTn) for each of the 6 times (T0-5) with the Skillings-Mack test, which is an equivalent of the Friedman test that can be used on a sample with missing or equal data (21). We then used the Mann–Whitney Wilcoxon test for paired data to make pairwise comparisons.

## 3 Results

The results are presented in Table 1.

**Table 1:** Pupil height (H) and length (L) measured in mm, before (Be) and after (Af) nose twitching.

		T0	T1	T2	T3	T4	T5
H	Be (mm)	11.73 [9.22; 13.91]	8.44 [5.68; 12.04]	16.95 [14.73; 18.77]	17.52 [16.02; 19.98]	16.80 [16.45; 17.80]	16.02 [15.20; 18.63]
	Af (mm)	12.09 [10.47; 14.52]	11.09 [7.97; 14.3]^*^	17.73 [15.30; 19.13]	17.16 [15.80; 19.59]	17.30 [15.84; 18.16]	17.09 [14.80; 18.63]
	PSV (%)	6.48 [−5.98; 19.02]	38.79 [16.01; 47.09] ^§^	3.24 [0.2; 9.82]^$^	1.02 [−0.48; 6.68]	−0.47 [−3.75; 5.37]	0.71 [−3.65; 6.16]
L	Be (mm)	17.09 [16.05; 19.67]	16.45 [14.80; 18.66]	19.66 [18.45; 20.41]	19.09 [18.02; 20.99]	17.88 [17.31; 19.45]	17.88 [16.30; 19.27]
	Af (mm)	19.52 [18.74; 21.40]^*^	18.31 [17.20; 20.52]^*^	20.31 [19.34; 21.17]	19.88 [19.20; 20.63]	17.80 [17.02; 18.95]	18.16 [16.48; 20.02]
	PSV (%)	12.49 [3.23; 17.67]^#^	9.63 [4.88; 16.21] ^#^	3.38 [0.72; 7.8]	0.82 [−2.88; 3.62]	−1.19 [−4.46; 2.34]^§^	2.91 [−6.06; 7.4] ^§^

Percentage in variation in pupil size (PSV) on awake horse (T0), 30 min after acepromazine administration (T1), after romifidine administration (T2), after morphine administration (T3), after ketamine and diazepam administration (T4) and during maintenance of anesthésia with an expired fraction of sevoflurane maintained at 2% (T5). Results are expressed as median and interquartile range [Q1-Q3]. *Significant difference between before and after nose twitching at Tn for H or L. ^#^ Significant difference between PSVL vs. PSVH at Tn. ^§^ Significant difference between PSVTn vs. PSVT0. ^$^ Significant difference between PSVTn vs. PSVTn-1.

Fourteen horses were finally recruited (Figure 1). They were referred to the hospital for various elective surgeries: castration, arthroscopy, electrochemotherapy, bursoscopy, removal of fragments of osteochondritis dissecans, or bone sequestration. The cohort of horses consisted of four mares, three geldings, and seven stallions and had a mean age of 5.6 (±3.7) years.

### 3.1 Effect of the nose twitch at each measurement time

Before application of the nose twitch and administration of any molecule, the pupil was 17.09 [16.05; 19.67] mm in length (LT0Be) and 11.73 [9.22; 13.91] mm in height (HT0Be).

In non-sedated horses (T0), the application of the nose twitch induced a significant increase in pupil length (*p* = 0.004), while pupil height increased non-significantly (*p* = 0.14). PSVLT0 and PSVHT0 were not significantly different (*p* = 0.36).

Thirty minutes after acepromazine administration (T1), pupil length and height increased significantly after positioning the nose twitch (*p* = 0.016 and *p* < 0.001 respectively). PSVHT1 was significantly higher than in PSVLT1 (*p* = 0.025).

Ten minutes after administration of romifidine (T2), the nose twitch had no significant effect on pupil height and length (*p* = 0.13 and *p* = 0.07 respectively). PSVHT2 was not different from that in PSVLT2 (*p* = 0.58).

Five minutes after morphine administration (T3), pupil length and height did not increase significantly after the nose twitch (*p* = 0.13 and *p* = 0.78 respectively). PSVLT3 and PSVHT3 were not significantly different (*p* = 0.19).

After induction (T4), pupil size did not vary significantly in length (*p* = 0.43) or height (*p* = 0.96) after the nose twitch was fitted. PSVLT4 was not significantly different from PSVHT4 (*p* = 0.73).

When the horses were anesthetized with a FeSevo of 2% (T5), pupil length and height did not vary significantly (*p* = 0.59 and *p* = 0.67 respectively) after the nose twitch was fitted. PSVLT5 and PSVHT5 did not differ (*p* = 0.95).

Two horses were not measured immediately after induction. These horses required more time to settle on the operating table. As a result, the horses had a FeSevo of 2% at the time of measurement. These measurements were considered for time T5. The results described below for time T4 are summarized based on twelve horses instead of the initial fourteen.

### 3.2 Percentage of pupil size variation (PSV) over time

The PSVL was significantly different overall between the different time periods (*p* = 0.005). However, a two-by-two comparison in chronological order did not reveal any significant difference in pupil length variation between the separate times. The only significant difference concerns PSVL at T4 and T5 compared to PSVL at T0 (*p* = 0.001; *p* = 0.03 respectively).

The PSVH was also significantly different overall between the separate times (*p* = 0.005). PSVH was higher at T1 than at T0 (*p* = 0.04). It was also significantly higher at T1 than at T2 (*p* < 0.001).

### 3.3 Pupil size variation over time

The variation in pupil height and length before the nose twitch application was significantly different overall between the various times (*p* < 0.001; *p* < 0.001 respectively). Romifidine induced mydriasis, shown by a significant increase in height and length (HT2Be versus HT1Be and LT2Be versus LT1Be) (*p* < 0.001; *p* < 0.001 respectively). Height and length remained constant until the end of the protocol: HT3Be versus HT2Be and LT3Be versus LT2Be (*p* = 0.31; *p* = 0.05); HT4Be versus HT3Be and LT4Be versus LT3Be (*p* = 0.34; *p* = 0.97); HT5Be versus HT4Be and LT5Be versus LT4Be (*p* = 0.16; *p* = 0.14).

## 4 Discussion

This study was carried out on a group of fourteen healthy horses undergoing anesthesia, and showed that twitching the nose produced a pupillary dilation in the awake and the sedated horse with acepromazine. During premedication and general anesthesia induction, this dilation was inhibited by the administration of romifidine and throughout the rest of the anesthesia protocol. In addition, we observed that these molecules had a direct effect on pupil size.

The pupil size values measured in our study are compatible with pupillometric measurements obtained by photography in a study evaluating the mydriatic effect of tropicamide 1% on the horse eye (22).

This study demonstrated that applying the nose twitch resulted in an increase in pupil length in conscious non-sedated horses, triggering a PDR similar to the response observed in the human pupil to pain (11). We indeed assumed that the nose twitch could serve as a painful stimulus. The nose twitch was used to create a reliable, repeatable, and reproducible acute painful/nociceptive stimulus whether the horse was conscious and standing or lying down under general anesthesia. We aimed to standardize the stimulus by having the same person perform the manipulation, using the same nose twitch and the same technique on each horse. However, this pain model cannot be extrapolated to spontaneous pain or chronic pain.

Nevertheless, its mechanism remains controversial and it is not one of the nociceptive stimuli validated for use in horses. The controversy surrounding this method arises due to the description of various mechanisms of action for the nose twitch. Different studies have observed that this method produces pain in the upper lip potentially raising pain tolerance during manipulation of other body parts (23, 24). Conversely, other research has observed an analgesic effect of this method, demonstrating an increase in endogenous β-endorphins, which could enhance pain tolerance (25). It seems difficult to conclude that nose twitch would induce an analgesic effect by the mere argument of a release of β-endorphins. In fact, it has been shown that the release of β-endorphins is a response to a painful or stressful stimulus (26). It has also been hypothesized that the nose twitch could have a deterrent effect, since, when applying this method, if the horse moves, it will experience pain and therefore try to avoid moving (27). Nevertheless, we cannot certify that its effect was effectively and sufficiently painful/nociceptive during our study. A validated scale for an induced pain model, such as the Equine Pain Face, could have been used; this would have allowed for a comparison of the results with pupil dilation.

Our primary expectation was an increase in pupil height rather than length, since radial muscle fibers are more developed on the vertical axis of the horse’s iris dilator muscle (28). Despite the trend observed, the small number of horses studied could explain why the height was not significantly different before and after the application of the nose twitch. The stress, which raised the sympathetic tone, may also have led to pupillary dilation before the application of the nose twitch. Indeed, the horse’s stress following the operator’s first approach could have led to an increase in pupil size before the nose twitch was placed, affecting the first measurement and therefore the amplitude of the dilation induced by the stimulus. Six of them were between two and 3 years old and three of them were admitted for castration. Thus, the approach of the operator and the manipulations carried out at T0 could have been stressful for these young horses with stallion-like behavior. In addition, our measurements showed some variability, which could be explained by individual-dependent stress.

It is interesting to note that the pupil size before the application of the stimulus in the horse tranquillized with acepromazine was smaller than in the awake horse. This may be the result of acepromazine-induced miosis. Acepromazine is an inhibitor of adrenergic and dopaminergic receptors (29). In dogs, the administration of acepromazine causes miosis (30).

Several mammalian species have adrenergic receptors at the neuromuscular synapses of the iris dilator muscle (28). We can hypothesize here that acepromazine inhibited the adrenergic receptors present in these synapses, leading to the relaxation of the iris dilator muscle. Pupil size was then solely dependent on the iris sphincter muscle, the tonus of which resulted in a decrease in the initial pupil size measured by the pupillometer. Another possible explanation is that under acepromazine-induced tranquillization, the pupil would have retained its normal size on approaching the pupillometer, whereas it would have dilated due to the stress induced by the approach of the machine in the non-sedated horse as mentioned above.

In horses tranquilized with acepromazine, the stimulus led to a more marked increase in pupil height than in pupil length. In addition, the pupillary dilation was greater than in non-sedated horses. Acepromazine therefore seems to potentiate the pupillary dilation induced by the stimulation, perhaps because the pupil size was initially reduced compared to that of an awake horse. The effect of acepromazine is reported to be paroxysmal 15 min after intravenous administration and may last for up to 2 h after injection (31). The thirty-minute gap between the administration of acepromazine and the measurement at T1 means that these measurements can be taken under the action of acepromazine, free from individual variability.

Romifidine appeared to inhibit the pupillary dilation in response to the stimulus. Romifidine is an α2-agonist used in horses for its sedative and analgesic properties (32–34). In humans, α2-agonists such as clonidine or dexmedetomidine reduce pupil size and inhibit the PDR (35, 36). In animals, however, the effects of α2-agonists vary between species. Clonidine causes mydriasis in animals (35). Dexmedetomidine causes miosis in dogs (30) and mydriasis in cats (37). In our study, intravenous injection of romifidine resulted in mydriasis. The mydriasis, already present before applying the nose twitch may have limited the pupillary dilation after its application. Nor can we rule out the possibility that the analgesic effect of romifidine contributed to the reduction in the pupillary dilation.

Five minutes after morphine administration, the painful stimulus did not induce a significant pupillary dilation. Opioids have been shown to reduce PDR in humans in a dose-dependent manner (10, 38). This reduction in pupillary dilation for the same given nociceptive stimulus was achieved within 5 min after administration of an opioid bolus in anaesthetized humans (39). In dogs, morphine induces miosis by exciting the pupil constrictor nuclei (28). On the other hand, in cats, morphine not only has the same effect but also triggers the release of catecholamines, thereby stimulating the sympathetic system and causing mydriasis (28). In horses, the iris sphincter muscle has muscarinic receptors and is blocked by cholinergic antagonists. The iris dilator muscle has adrenergic receptors that could cause contraction of this muscle and pupil dilation. To our knowledge, no study has demonstrated the composition and distribution of these receptors in the horse iris (40, 41). Therefore, it is difficult to conclude on the specific effect of morphine on the horse’s pupillary dilation. Nevertheless, we cannot exclude that the effect of romifidine (mydriasis) was still present and may have inhibited the reflex.

After induction of anesthesia, the pupillary dilation was still inhibited. These results are contrary to those observed in humans. One study showed that the PDR persisted following tetanic stimulation in a sample of twenty-four children whose anesthesia had been induced by a bolus of ketamine (42). As for diazepam, its influence on PDR has never been studied to our knowledge. However, it does not appear to influence the control of iris muscles (43). Pupillometry has been used to measure PDR in patients whose anesthesia was maintained with sevoflurane (44) even though pupillary reactivity may be reduced compared with intravenous propofol anesthesia (45). We cannot exclude that the mydriatic effect of romifidine and morphine was still present at these measurement dates.

### 4.1 This study has several limitations

First, it was chosen to work on horses free of eye conditions, devoid of pain, not receiving drug treatment, and admitted for elective surgery. This point made it possible to exclude altered reactivity of the pupil, any influence of molecules other than those of our anesthetic protocol, the presence of pain before the first measurement at T0, and to have only the application of the nose twitch as only painful stimulation. Our results therefore only apply to this type of patient.

Second, it can take several minutes for the horse’s eye to adapt to a new light (46). The measurements were conducted in three different rooms (the hospitalization stall, the induction stall, and the operating room). The measurements were made after an adaptation period; however, the luminosities were different. Even though, ambient light does not affect PDR in humans (8), but the horse’s second eye was not hidden during our measurements possibly exhibiting a pupillary light reflex.

Third, the study only involved fourteen horses. The power calculation shows that the findings can be applied to a population of healthy, calm-behaved horses similar to our study group. Nevertheless, the conclusions drawn from our results cannot be generalized to horses in general. The study could hardly be randomized or blinded, nevertheless, photos of the pupillometer screen were taken to validate the measurements with observers unaware of when the photo was taken.

Fourthly, any kind of clinical pain is very different from what can be produced by a nose twitch on the upper lip. Nevertheless, we did not intend to extrapolate experimental pain to clinical pain. We aimed to use a stimulus that was reliable, consistent, and reproducible to create a pain model and to observe the pupil reaction. Initially, we wanted to know whether the pupillometer could be used under the same conditions as in humans, i.e., to check that the level of analgesia is sufficient in response to an acute nociceptive stimulus (i.e., surgery acts under general anesthesia). In other words, to use the pupillometer to measure the quality of per-operative analgesia and not as a measure of pain in general. However, initial trials have shown us that it is difficult to measure per-operative pupillary dilatation in horses. Indeed, even if the eye remains central under anesthesia in this species (unlike that of small animals which tilts) it presents complete mydriasis masking any further pupillary dilatation. We therefore wanted to know whether the horse’s eye dilates under the effect of acute pain and, if so, at what point in the anesthesia protocol this dilation was no longer detectable. That’s why we have chosen a stimulus that was as reproducible as possible, but also practical to use on an awake non-sedated standing horse, then standing and sedated horse, then lying down anaesthetized horse. The aim was also to use the same stimulus throughout all stages of anesthesia (premedication, induction, maintenance).

At last, a comparison group with another means of detecting pain and also nociception (since the notion varies throughout the protocol) could have been interesting even essential if the aim was to validate the use of the pupillometer to measure pain. There are not many validated pain scales in equine species. The Equine Pain Face was validated with two experimentally induced pain models (a tourniquet on the antebrachium and topical application of capsaicin). We could indeed have used this scale to compare with the results of pupillometry. However, as the stimulus was not the same, the validity of this grid would have been questionable. Furthermore, every scale is subject to uncertainties (even if validated, they are validated under very precise conditions), and comparing two imperfect systems cannot lead to a conclusion.

## 5 Conclusion

These elements highlight that pupillary dilation, in response to a stimulation considered painful, exists in horses. Further studies are needed to evaluate the specific effects of α2-agonists and opioids on pupil size and dilation in horses. Furthermore, it would be necessary to be able to overcome the mydriasis observed intraoperatively to use the pupillary dilation to guide anti-nociceptive treatment. This is done using a calibrated nociceptive stimulus (such as tetanic stimulation in humans) and validated in horses. The study of the effects of antinociception guided by pupillometry on the quality of recovery and postoperative complications would then be very interesting.

## Data availability statement

The original contributions presented in the study are included in the article/supplementary material, further inquiries can be directed to the corresponding author.

## Ethics statement

The animal studies were approved by Ethical Committee of the National Veterinary School of Lyon (Number 2234, April 12th, 2022). The studies were conducted in accordance with the local legislation and institutional requirements. Written informed consent was obtained from the owners for the participation of their animals in this study.

## Author contributions

CM: Writing – original draft, Writing – review & editing, Conceptualization, Data curation, Investigation, Methodology. SK: Writing – original draft, Writing – review & editing, Conceptualization, Data curation, Formal analysis, Investigation, Methodology. MA: Writing – review & editing, Conceptualization, Data curation, Investigation, Methodology. KP: Supervision, Writing – original draft, Writing – review & editing, Conceptualization, Data curation, Formal analysis, Funding acquisition, Investigation, Methodology, Validation.
